# Implementation and evaluation of the Helping Babies Breathe curriculum in three resource limited settings: does Helping Babies Breathe save lives? A study protocol

**DOI:** 10.1186/1471-2393-14-116

**Published:** 2014-03-26

**Authors:** Akash Bang, Roopa Bellad, Peter Gisore, Patricia Hibberd, Archana Patel, Shivaprasad Goudar, Fabian Esamai, Norman Goco, Sreelatha Meleth, Richard J Derman, Edward A Liechty, Elizabeth McClure, Waldemar A Carlo, Linda L Wright

**Affiliations:** 1 Mahatma Gandhi Institute of Medical Sciences, Sewagram, India; 2 KLE’s Jawaharlal Nehru Medical College, Belgaum, India; 3 Moi University, Eldoret, Kenya; 4 Massachusetts General Hospital, Boston, MA, USA; 5 Lata Medical Research Foundation, Nagpur, India; 6 RTI International, Research Triangle Park, Durham, NC, USA; 7 Christiana Care, Newark, DE, USA; 8 Indiana University, Indianapolis, IN, USA; 9 University of Alabama at Birmingham, Birmingham, AL, USA; 10 Eunice Kennedy Shriver National Institute of Child Health and Human Development, Bethesda, MD, USA

**Keywords:** Neonatal mortality, Perinatal mortality, Asphyxia, Stillbirth, Helping Babies Breathe, Resuscitation, Bag and mask ventilation, ≥1500 grams

## Abstract

**Background:**

Neonatal deaths account for over 40% of all under-5 year deaths; their reduction is increasingly critical for achieving Millennium Development Goal 4. An estimated 3 million newborns die annually during their first month of life; half of these deaths occur during delivery or within 24 hours. Every year, 6 million babies require help to breathe immediately after birth. Resuscitation training to help babies breathe and prevent/manage birth asphyxia is not routine in low-middle income facility settings. Helping Babies Breathe (HBB), a simulation-training program for babies wherever they are born, was developed for use in low-middle income countries. We evaluated whether HBB training of facility birth attendants reduces perinatal mortality in the *Eunice Kennedy Shriver* National Institute of Child Health and Human Development’s Global Network research sites.

**Methods/design:**

We hypothesize that a two-year prospective pre-post study to evaluate the impact of a facility-based training package, including HBB and essential newborn care, will reduce all perinatal mortality (fresh stillbirth or neonatal death prior to 7 days) among the Global Network’s Maternal Neonatal Health Registry births ≥1500 grams in the study clusters served by the facilities. We will also evaluate the effectiveness of the HBB training program changing on facility-based perinatal mortality and resuscitation practices. Seventy-one health facilities serving 52 geographically-defined study clusters in Belgaum and Nagpur, India, and Eldoret, Kenya, and 30,000 women will be included. Primary outcome data will be collected by staff not involved in the HBB intervention. Additional data on resuscitations, resuscitation debriefings, death audits, quality monitoring and improvement will be collected. HBB training will include training of MTs, facility level birth attendants, and quality monitoring and improvement activities.

**Discussion:**

Our study will evaluate the effect of a HBB/ENC training and quality monitoring and improvement package on perinatal mortality using a large multicenter design and approach in 71 resource-limited health facilities, leveraging an existing birth registry to provide neonatal outcomes through day 7. The study will provide the evidence base, lessons learned, and best practices that will be essential to guiding future policy and investment in neonatal resuscitation.

**Trial registration:**

Trial registration ClinicalTrials.gov Identifier: NCT01681017

## Background

Neonatal deaths account for over 40% of the under-5 years old deaths and are increasingly critical for accelerating progress for Millennium Development Goal 4 (MDG4) [1,2]. Neonatal deaths have been reduced from 4.6 to 3.3 million in two decades, but the average annual rate of reduction of the neonatal mortality rate is 1.7%, compared to 2.2% in postnatal and child mortality and 2.3% in maternal mortality. Five large countries account for over 50% of neonatal deaths—India alone has over 900,000 newborn deaths and one in every four newborn deaths worldwide is an Indian neonate. Africa is being left further and further behind with an average annual rate of neonatal mortality reduction under 1% per year. Without more action for newborn survival in Africa, it will be over 150 years before African newborns have the same chance of survival as a baby born today in Europe or North America [1].

The *Eunice Kennedy Shriver* National Institute of Child Health and Human Development (NICHD) Global Network for Women’s and Children’s Health Research (Global Network) was initiated to conduct sustainable, high-impact research targeted at the major health problems of mothers, infants, and young children in developing countries. The investment in the Global Network sites over 11 years has resulted in uniquely qualified study venues that are suited to successfully carry out studies such as those proposed here [3,4].

### Interventions to prevent neonatal asphyxia

Resuscitation training using a standard national program began in the United States with the 1987 publication of the American Academy of Pediatrics (AAP) Neonatal Resuscitation Program [5]. Since then, few studies have attempted to quantify the impact of resuscitation training on newborn outcome [6,7]. The Bang et al. study (1993–1998) in Gadchiroli, India, trained female Community Health Workers to do home visits to provide health education to women, monitor neonates, and manage asphyxia, preterm birth and neonatal infection. They reported a 47.5% decrease in the case fatality in neonates with severe asphyxia from 39 to 20% (p < 0.07) and asphyxia-related mortality by 65%, from 11 to 4% (p < 0.02) [6]. Carlo et al. studied resuscitation training of midwives in low-risk, urban community health clinics in Zambia and documented reductions in mortality within the first 24 hours after birth: 7.8 deaths per 1000 live births among newborns delivered by intervention midwives compared with 19.9 per 1000 among newborns delivered by control midwives (RR 0.40, 95% CI 0.19 to 0.83) [7]. NICHD’s Global Network conducted the first randomized controlled trial of resuscitation training (The FIRST BREATH Trial) in 7 countries [3]. The FIRST BREATH Trial included all deliveries ≥1500 grams and aimed to train all birth attendants (BAs) (including BAs, Traditional Birth Attendants [TBAs], and families) to provide prompt essential newborn care (ENC) and resuscitation [3]. The potential reduction in neonatal mortality was estimated to be as high as 30 to 40% [8].

In the interim between the Global Network’s FIRST BREATH trial and the implementation of the HBB study, the circumstances of birth have changed: the proportion of facility-based deliveries have almost doubled from 40 to 80% in some of the most vulnerable states in India [9,10], and some countries now allow only facility BAs to be trained to encourage delivery in health facilities [11].

### Rationale for the study

Helping Babies Breathe (HBB), a new training program to resuscitate neonates wherever they are born, represents a collaboration between the American Academy of Pediatrics (AAP), NICHD’s Global Network, the Laerdal Medical, and global partners [12]. Pilot tests of the program were conducted in India, Pakistan, Kenya, Tanzania, and Bangladesh in 2009 and 2010 [13,14]. The United States Agency for International Development (USAID) then spearheaded the development of a Global Development Alliance, including AAP, NICHD, Laerdal Medical, and other partners to support the global implementation of the HBB program [13].

The HBB package of training materials has not been formally tested in a large study using a common design, materials, and training model. However, a number of studies have evaluated the impact of neonatal care and resuscitation training for physicians, nurses, and other BAs on their skills and knowledge [14-17]. These studies have suggested that BAs at different levels of skill can be trained to effectively resuscitate newborns; however, the amount of retraining required to retain the skills is unknown and may vary by setting. In 2011, USAID invited NICHD’s Global Network to evaluate the impact of HBB. Because rollout of HBB had started, the critical window of opportunity to demonstrate whether HBB saves neonatal lives was narrow, therefore a pre-post study was developed to use the Global Network’s high-quality, existing Maternal and Newborn Health Registry (MNH Registry) in Global Network sites in India and Africa where the neonatal mortality rate is high [4]. The advantages of the established MNH Registry include the accuracy and completeness of the data and the availability of “pre-intervention” data in locations where BAs had received limited previous resuscitation training.

A crucial component of HBB is training BAs about the importance of intervening with the appropriate resuscitation *if needed* within the first 60 seconds (the Golden Minute) after birth. By focusing on the timely delivery of the essential interventions of drying, warmth, stimulation to breathe and bag and mask ventilation (if required), most babies who are not breathing at birth can be saved. The HBB training materials are of high quality—they use multiple reinforcing modalities (color, format, cartoon-like illustrations, and three simple care paths); focus on critical decision points; and give a specific time limit by which ventilation should be established (the Golden Minute). The teaching materials, which include a realistic neonatal simulator (NeoNatalie), allow trainers to simulate and provide practice in the management of serious birth complications with a model that feels like a depressed newborn versus a fresh stillbirth. This extra practice/training should assist in the maintenance of a high skill level among BAs who have a limited number of deliveries. Quantifying the impact of a program like HBB is challenging, but essential to guide future policy and investment.

## Methods/design

This two year prospective pre-post study will evaluate the impact of a package of interventions, including HBB and essential newborn care training [18] and provision of equipment, on the perinatal mortality rate (lives saved) in the participating clusters of the Global Network before and after implementation in facilities that serve the clusters. In addition to quantifying the impact of HBB as a public health intervention pre- and post-training, the study will also provide data on the effectiveness of the training and monitoring program and its impact on all perinatal mortality and resuscitation practices in the participating facilities. The intervention will consist of three major components: selection of the Master Trainers (MT) and Birth Attendants (BA); training them in HBB/ENC and monitoring activities, and implementing quality improvement (QI) activities (Additional file 1: Panel 1).

### Site description

The HBB/ENC implementation study will be conducted in three Global Network research units, located in Belgaum and Nagpur, India, and Eldoret, Kenya that have conducted common clinical research for three years under an NICHD cooperative agreement (the Global Network). Data from the MNH Registry, a prospective population-based registry (initiated in 2008) of all pregnancy and neonatal outcomes (including vital status through 42 days, cause of perinatal death and neonatal resuscitation) in 52 defined geographic clusters with 300–500 births per year, will be used to select the facilities where the HBB training will be conducted.

### Selection of the intervention facilities

The study will be conducted in a select number of health facilities that represent a substantial portion of facility births in the three research units’ clusters (44% of Belgaum, 41% of Nagpur, and 24% of Kenya’s facility births), based on the 2010 MNH Registry data. The facilities must also provide 24-hour coverage for deliveries 7 days/week and have a perinatal mortality rate of 30 per 1,000 deliveries in the pre-study period. Women who reside in and/or deliver in the study clusters will be approached for enrollment in the MNH Registry by separate staff; the primary outcome will be calculated using all delivery data from the MNH Registry during the study period. Addition of other facilities would not have significantly increased the coverage of MNH facility births.

### The intervention

The intervention will consist of three major components: selection of the MTs and BAs for training; training; and quality improvement QI and monitoring activities. Details of the components include: (1) selection of MTs, Facilitators and Learners, (2) country-level training of MTs in the HBB/ENC curricula, (3) facility-level training of BAs in the HBB/ENC curricula (4) periodic refresher training and evaluation during the implementation of the training program (5) QI, including regular observation of deliveries in participating study health facilities, unannounced observation of deliveries or HBB skills (using a neonatal simulator if no deliveries are available), resuscitation debriefings, perinatal death audits, daily bag and mask ventilation practice, daily check of cleanliness and availability of resuscitation equipment, and monthly monitoring reports, (6) biweekly calls with research unit HBB coordinator staff, data center, and NICHD staff followed by discussions with facility MTs and BAs.

### Selection of master trainers and birth attendants

The appropriate selection of MTs and BA is important to ensure fidelity of the intervention/training and the maintenance of resuscitation skills over time. The sites will select the country level MTs and potential new MTs based on criteria outlined in the HBB Implementation Guide [12]. The AAP will provide technical assistance to identify HBB training best practices and assist the HBB central core staff (RTI International and NICHD) in developing two tiers of HBB training workshops: central level MT courses at each study site (Belgaum, Nagpur, and Eldoret); and facility level BA courses.

### Country-level training of Master Trainers in the HBB/ENC curricula

Three country-level HBB MT workshops will be held, one each in Eldoret, Belgaum, and Nagpur. The country MT teams will include 2 teams of 3 AAP HBB MTs paired with 3 site HBB MTs from Belgaum, Nagpur, and Eldoret, including physicians, midwives, and nurses, to provide a ratio of 6 learners to one MT. Their goal will be to co-lead a 4-day, intense, hands-on workshop to provide a large cohort of new MTs for Nagpur, Belgaum, and Eldoret (at least one MT for every facility), including pre-post training assessment (Additional file 1: Panel 2). This flattened training cascade was designed to preserve the integrity of the intervention, allow rapid start up, minimize training costs, and provide at least one MT for each facility.

### Facility-level training of BAs

The newly trained MT cohort will teach the BAs in facilities in 3-day hands-on workshops using the same ratio of 6 trainees to one new MT with pre-post assessment. Staff turnover will require HBB training on an individual basis.

### Refresher training

Six months following the completion of initial HBB training, the site research teams will conduct refresher training courses for active BAs that received prior HBB training. The refresher training courses will include the use of the HBB knowledge and skill videos developed by the AAP and evaluations to examine retention and progress with skills development. QI and monitoring activities are included as part of the study (Additional file 1: Panel 1).

### Providing a model of positive supervision, problem solving, accountability and team building

The HBB coordinator for each research unit will develop an infrastructure to encourage timely, accurate data gathering, and problem solving by encouraging the facility staff to practice bag and mask ventilation, encourage development of group improvement goals, and maintain standardized delivery room birth records that will provide the basis for QI and monitoring activities. The records will be reviewed on a biweekly call between the HBB site coordinator and staff, RTI HBB staff, and NICHD. Minutes of the bi-weekly conference calls and recommendations will be circulated to the sites for discussion with facility staff.

### Data collection and management

Data for the primary outcome will be derived from the independent data collection from the MNH Registry as described above and collected by the MNH Registry staff. In addition, we will collect HBB-specific data to evaluate the HBB training, program and QI and monitoring activities in the facilities.

### Data analysis

The primary outcome is the difference in the perinatal mortality rate (fresh stillbirths or neonatal deaths prior to 7 days after birth) among births ≥1500 grams across the three participating clusters pre vs. post intervention. The pre-phase is defined as the 12-month period preceding the implementation of the intervention (the HBB facility training will be rolled out at different points in time). The post phase will be the 12-month period after the completion of the initial training of the MTs and BAs in each of the participating facilities. The primary analysis will focus on cluster-level variables, i.e., on measures aggregated at the cluster level for each of the 52 clusters, and the analyses described below will include data from all clusters at all research units.

The PMR will be estimated by dividing the sum of ≥1500 gram fresh still births and live births that die within the first 7 days after birth by the total number of ≥1500 grams live births and fresh stillbirths. The PMR will be estimated for each cluster for the pre HBB period and the post HBB period.

The primary outcome will be tested using a linear mixed model that incorporates both random cluster effect and fixed effects for time (pre- versus post HBB/ENC training and equipment). The random cluster effect accounts for the correlation between measures across time from the same cluster. The dependent variable will be the cluster-level perinatal mortality rate aggregated separately across the pre and post periods. A dichotomous variable (period) post versus pre HBB/ENC training and equipment implementation will be the fixed treatment effect. A contrast term will be used to test for differences in pre versus post perinatal mortality.

### Secondary analyses

Key secondary outcomes include facility measures: (1) difference in the rate of facility-based perinatal mortality among births ≥1500 grams, pre vs. post HBB/ENC implementation, again evaluated at the cluster level; (2) difference in the cluster-level rate of facility-based fresh stillbirths ≥1500 grams, pre vs. post HBB/ENC implementation; and (3) difference in mean number of resuscitations with bag and mask among births ≥1500 grams pre vs. post HBB/ENC implementation. These analyses will evaluate the changes in PNM, FSB and number of bag and mask resuscitations among MNH Registry patients at the facility level (approximately half of the facilities had HBB-trained BAs).

The analysis for all of the secondary mortality outcomes will also be tested using linear mixed models, with a structure comparable to that used for the primary analysis that incorporates both random cluster effect and fixed effects for time (pre versus post HBB/ENC training and equipment).

### Other secondary outcomes

Other secondary outcomes include the difference in mean scores on multiple-choice resuscitation knowledge tests, observed bag and mask skills, and tests of resuscitation management using the Objective Structured Clinical Exams (OSCE) A and B. The hypothesis tested is that the training increases BAs’ skills in adhering to the HBB protocol. A paired t-test will be used to test the hypothesis that the mean difference in score (post minus pre training) is greater than zero. Additionally, secondary analyses will evaluate the cluster and facility results over time.

### Sample size

#### Power estimates

Consistent with the analytic approach proposed for this study, estimates of the power associated with the clusters within the three Global Network sites for the HBB study were generated based on cluster-level analyses of the 2010 data from the MNH Registry for these three sites. The PMR for babies with birth weight of 1500 grams is approximately 25 per 1000 births. A 20% reduction in this case would result in a reduction of approximately 5 deaths per 1000. The standard deviation of PMR between clusters for the subset of babies with a birth weight of ≥1500 is approximately 10 with a correlation across time of about 0.3. The resulting estimate of the standard deviation for the difference in PMR across time in this population is approximately 12. Based on these assumptions, the study has a power of approximately 82% to detect a 20% reduction in PMR among births with a birth weight of ≥1500 grams.

### Ethical considerations

The AAP HBB and WHO ENC training programs utilize global standard of care approaches to survival of newborns. The HBB study protocol and informed consents were submitted and approved by the Institutional Review Boards of the participating clinical sites and RTI International, as the data center. The study was also reviewed and approved by the Indian Council of Medical Research for the Belgaum and Nagpur, and the Kenya Institutional Review Board. A written informed consent will be obtained from all learners trained and monitored in HBB and ENC skills.

The pregnant women and their neonates will participate in this study as part of the Global Network MNH Registry, which was also reviewed and approved by each site’s IRB. The consent obtained from the pregnant women who consented to be part of the MNH Registry covered all HBB study procedures.

## Discussion

The study will evaluate the effect of a HBB/ENC training and a QI and monitoring package on perinatal mortality using a large multicenter common design and approach in 71 resource-limited health facilities, leveraging an existing birth registry to provide neonatal outcome data through day 7. The study will provide the evidence base, lessons learned, and best practices that will be essential to guiding future policy and investment in neonatal resuscitation.

## Abbreviations

AAP: American Academy of Pediatrics; ANC: Antenatal care; BMV: Bag and mask ventilation; BA: Birth attendant; DR: Delivery room; ENC: Essential newborn care; GN: Global Network for Women’s and Children’s Health Research; HBB: Helping Babies Breathe; MNH: Maternal and Newborn Health; MT: Master trainer; MCQ: Multiple choice questionnaire; MDG4: Millennium Development Goal 4; MNH: Maternal Newborn Health; NeoNatalie: Realistic neonatal simulator; NICHD: *The Eunice Kennedy Shriver* National Institute of Child Health and Human Development; OSCE: Objective structured clinical evaluations; PMR: Perinatal mortality rate; QI: Quality improvement; TBA: Traditional birth attendants.

## Competing interests

The authors declare that they have no competing interests.

## Authors’ contributions

AB, LLW and PH wrote the first draft, revised and reviewed the final version; all co-authors provided input into the study protocol and reviewed the final manuscript. SM provided statistical expertise. All authors read and approved the final manuscript.

## Pre-publication history

The pre-publication history for this paper can be accessed here:

http://www.biomedcentral.com/1471-2393/14/116/prepub

## Supplementary Material

Additional file 1 **Panel 1.** Helping Babies Breathe Trial Quality Improvement and Monitoring Activities. **Panel 2.** Sample Training Agendas. Click here for file
